# The effect of Problem-Based Learning on Care Management skills: A quasi-experimental study*

**DOI:** 10.1590/1518-8345.6272.3867

**Published:** 2023-03-27

**Authors:** Luis Angel Benítez-Chavira, Rosa Amarilis Zárate-Grajales, María Guadalupe Moreno-Monsiváis, Cecilia Xochitl Vite-Rodríguez, Carlota Mercedes Hernández-Rosales, Carlos Emmanuel Brito-Carbajal

**Affiliations:** 1 Universidad Nacional Autónoma de México, Escuela Nacional de Enfermería y Obstetricia, Ciudad de México, CDMX, México; 1 Universidad Autónoma de Nuevo León, Facultad de Enfermería, Monterrey, Nuevo León, México

**Keywords:** Problem-Based Learning, Education, Aptitude, Administration, Nursing, Mexico, Aprendizaje Basado en Problemas, Educación, Aptitud, Administración, Enfermería, México, Aprendizagem Baseada em Problemas, Educação, Aptidão, Administração, Enfermagem, México

## Abstract

**Objective:**

to assess the preliminary effect of Problem-Based Learning on Care Management skills.

**Method:**

a quasi-experimental pre- and post-test conducted with students attending the Bachelor’s Degree in Nursing offered by an educational institution. The sample was comprised by 29 (Experimental Group) and 74 (Control Group) students. The Experimental Group solved four scenarios under the Problem-Based Learning method with the 7 steps proposed by McMaster University, in a Care Management program in distance mode. The self-reporting instrument assessed the pre- and post-test Care Management skills in both groups. Mean values were obtained and descriptive and inferential statistics were performed (Student’s t, paired t, linear regression).

**Results:**

the Experimental Group obtained higher scores in analytical, action-related and global skills than the Control Group (p<0.05). No differences were recorded in interpersonal skills or in use of the information. The Control presented no significant differences before and after usual teaching, whereas differences were in fact reported in the Experimental Group (p<0.05).

**Conclusion:**

despite the fact that there is little evidence on the development of Nursing Care Management skills, the current study shows that Problem-Based Learning is an effective and significant method in remote education.

## Abstract (es)

**Objetivo:**

evaluar el efecto preliminar del Aprendizaje Basado en Problemas en habilidades de Gestión del Cuidado.

**Método:**

cuasiexperimental pretest-postest, con alumnos de la carrera de Licenciatura en Enfermería de una institución educativa. Muestra de 29 alumnos (Grupo Experimental) y 74 (Grupo Control). El Grupo Experimental solucionó cuatro escenarios bajo el método de Aprendizaje Basado en Problemas con los 7 pasos propuestos por la Universidad de McMaster, en un programa de Gestión del Cuidado en modalidad a distancia. El instrumento autoinformado evaluó las habilidades de Gestión del Cuidado *pretest*y *postest*en ambos grupos. Se obtuvieron valores medios y se realizó estadística descriptiva e inferencial (t de Student, t pareada, regresión lineal).

**Resultados:**

el Grupo Experimental obtuvo puntuaciones más elevadas en habilidades analíticas, de acción y globales que el Grupo Control (p<0,05). No se registraron diferencias en las habilidades interpersonales ni en el uso de la información. El Grupo Control no presentó diferencias significativas antes y después de la enseñanza habitual, mientras que en el Grupo Experimental sí se reportaron diferencias (p<0,05).

**Conclusión:**

a pesar de que existe poca evidencia en el desarrollo de habilidades de Gestión del Cuidado en Enfermería, el presente estudio demuestra que el Aprendizaje Basado en Problemas es un método efectivo y significativo en educación a distancia.

## Abstract (pt)

**Objetivo:**

avaliar o efeito preliminar da Aprendizagem Baseada em Problemas nas habilidades de Gestão do Cuidado.

**Método:**

pré e pós-teste quase experimental, realizado com alunos do curso de Bacharelado em Enfermagem de uma instituição de ensino. A amostra foi composta por 29 (Grupo Experimental) e 74 (Grupo Controle). O Grupo Experimental resolveu quatro cenários sob o método de Aprendizagem Baseada em Problemas com os 7 passos propostos pela McMaster University, em um programa de Gestão do Cuidado na modalidade à distância. O instrumento de autorrelato avaliou as habilidades de Gestão do Cuidado pré e pós-teste em ambos os grupos. Valores médios foram obtidos e estatísticas descritivas e inferenciais foram realizadas (t de Student, t pareado, regressão linear).

**Resultados:**

o Grupo Experimental obteve escores mais elevados em habilidades analíticas, de ação e globais do que o Grupo Controle (p<0,05). Não foram registradas diferenças nas habilidades interpessoais ou no uso da informação. O Grupo Controle não apresentou diferenças significativas antes e depois do ensino usual, enquanto as diferenças foram de fato relatadas no Grupo Experimental (p<0,05).

**Conclusão:**

apesar de haver poucas evidências sobre o desenvolvimento de habilidades de Gestão do Cuidado de Enfermagem, o presente estudo mostra que a Aprendizagem Baseada em Problemas é um método eficaz e significativo na educação à distância.

**Highlights**

(1) PBL develops management skills (analytical and action-related).

(2) In the remote modality, PBL is a possible effective strategy in terms of management.

(3) The type of students, their performance and experience in PBL might predict their skills.

(4) It is necessary to train the professors in PBL for it to achieve its objective in the students.

## Introduction

In the 21 ^st^ century, Nursing has positioned itself as one of the main disciplines that contribute to improving the health of the individual, the family and the population, due to its ability to perform the multiple roles and responsibilities required by current health systems: care, teaching, research, politics and, in recent years, in terms of management ^( 1- 2)^ . 

Care Management has been defined since 1996 as a heuristic process that implies a dialectical relationship between Nursing management and care knowledge, articulated with the help of administrative sciences to mobilize human and environmental resources to facilitate safe and timely care ^( 3- 4)^ . However, the constant social, political, economic and epidemiological changes force its teaching from the undergraduate level to ensure the development of managerial skills in new novice Nursing professionals ^( 5)^ , who should exercise the role of managers with specific skills to improve the quality of services and of care itself. 

In this regard, some authors point out the skills that an undergraduate student should have in terms of management, in addition to providing background on which should be attributable to novice or beginner Nursing professionals. As an example, in Bosnia and after a study in various health institutions ^( 6)^ , it was concluded that there are 4 main knowledge areas and basic skills that they should have, namely: 1) interpersonal, 2) use of the information, 3) analytical, and 4) action-related. Following these arguments, a manager must know how to exercise leadership, work in a team, communicate openly and efficiently, lead health teams and users, have strategic thinking and make decisions (in a planned, organized, directed and controlled way) to improve quality of the health services ^( 7- 9)^ . 

Despite the evidence on the main skills that a manager must have, several authors have concluded that Nursing professionals and beginner-level students who assume the new role of Nursing managers and have to put their skills into practice, and that they often encounter dilemmas, doubts, conflicts and conceptual misunderstandings, leading to deficient care management, which negatively affects the outcomes in the patients. They also mention that some of the barriers are culture and organizational structure and lack of experience in the professional Nursing practice; as is the case with the government’s public policies and precarious conditions of the workforce; but, above all, lack of education, formal and continuous training in educational institutions and the quality with which it is taught ^( 10- 13)^ . 

In this context, there are important challenges for the development of management skills in educational institutions in order to have highly trained Nursing professionals with experience in management of health services ^( 14)^ . 

To achieve the aforementioned, it is necessary that the professors use didactic strategies to promote the teaching-learning process and contribute to the development of skills in the students ^( 15)^ and that these may be successfully used and adapted in the remote modality ^( 16)^ . Different studies carried out with Nursing students assert that the Problem-Based Learning method is one of the didactic strategies that contribute to the development of critical thinking, increase theoretical and practical knowledge, improve specific and self-directed skills and prepare students to face unknown challenges for their professional practice ^( 17- 19)^ . 

### Problem-Based Learning (PBL)

PBL is an educational strategy based on the use of real or fictitious problems as a starting point, mainly centered on the students. Since 1986, the McMaster University has conceptualized PBL as “the learning resulting from the process of working towards understanding or solving a problem” ^( 20)^ . The core value lies in employing a contextualized problem to motivate the students to actively seek the relevant knowledge using all the resources possible. Likewise, the PBL consists of posing a problem situation, where its construction, analysis and/or resolution constitute the central focus of the experience in the students, and where teaching consists of promoting the development of the inquiry and problem-solving process in an organized way ^( 21)^ . 

Review articles and meta-analyses carried out in Nursing and Medicine areas conclude that PBL increases students’ performance and exerts a positive effect on theoretical and practical test scores ^( 17- 18, 22)^ . It also contributes to the development of critical thinking, increases Nursing knowledge and self-directed skills required for clinical, management and leadership hospital settings ^( 19)^ ; however, there is little current evidence on the use of PBL to develop management skills in undergraduate students, although it is in fact suggested that this method should be implemented in Nursing programs to evidence its effectiveness ^( 23- 24)^ . 

Some studies, such as those carried out in South Africa, Brazil, Turkey, China and Japan, have shown that using the PBL method in management programs contributes to the development of interpersonal skills, especially communication skills ^( 25- 29)^ , leadership ^( 25, 28, 30- 32)^ , teamwork ^( 27, 30)^ and interpersonal relationships ^( 28- 29)^ . 

It also improves analytical skills in relation to having a strategic vision, making use of technology and statistics, as well as human and material resource planning skills ^( 25- 26, 29- 30, 32- 33)^ . It also develops skills in continuous improvement topics ^( 30, 34)^ . In the same way, action-related skills are developed, but to a lesser extent, and are associated with being able to establish, organize and evaluate goals/objectives and make timely and risk-free decisions in problematic situations ^(^^( 27, 29- 30, 32, 35)^ . Finally, the least developed skill is use of the information, such as collecting and analyzing the hospital environment data ^(^^( 30, 34)^ . 

Despite the fact that there is conclusive literature on the development of management skills with the use of PBL throughout the world, in Mexico there are few or no recent studies on the effect of using this type of methodology on students in the remote modality of the Bachelor’s Degree in Nursing to develop self-directed skills that are related to management (interpersonal, use of information, analytical and action-related) ^( 36)^ and that contribute in the future to provide a professional practice based on timely, safe and efficient care according to the individual needs of each patient and their own health institution. Therefore, it is important to carry out studies of this nature in order to increase knowledge about the positive effects of PBL in Nursing students and that the professors and mentors of programs related to management/administration use this strategy to successfully complete the teaching-learning process. 

Due to the above, this research is one of the first efforts in the training of students in Care Management in the country, as well as the first stage to recognize processes, strengths, weaknesses and limitations and, with this, evaluate the preliminary effect of Problem-Based Learning in Care Management skills in the remote modality among Nursing undergraduate students at a public educational institution from Mexico City.

## Method

### Design

This is a quasi-experimental study ^( 37- 39)^ (Experimental Group *vs.* Control Group) to evaluate the effect of the PBL methodology through the implementation and solution of problem-scenarios with a model (pre-test/ intervention/post-test), in the remote education modality on Care Management skills. This study followed the TIDieR ( *Template* *for* *Intervention* *Description* *and* *Replication*) guidelines for the presentation of intervention studies and as a measure for future replications. 

### Period

The study was conducted during the 2022-1 school cycle: it was initiated on August 9 ^th^, 2021, and ended on October 30 ^th^, 2021, during the COVID-19 pandemic. During such period, education was at a distance due to the temporary closure of educational institutions and the suspension of clinical practices in health institutions. 

### Population and sample

The research participants were students attending the Nursing Bachelor’s Degree enrolled in the Care Management Program, the seventh semester (7/8) of the National Nursing and Midwifery School ( *Escuela Nacional de Enfermería y Obstetricia*, ENEO) belonging to the National Autonomous University of Mexico ( *Universidad* *Nacional* *Autónoma de México*, UNAM) in 2021. 

The ENEO students’ academic profile is structured into 3 training cycles: Basic Care Fundamentals, which introduces the students to philosophical, biological and social sciences for the understanding of care (first and second semesters); Human Life Cycle, which promotes clinical skills for care (third, fourth, fifth and sixth semesters); and Collective Health, which delves into knowledge and research methods and care management to design intervention models that meet care needs (seventh and eighth semesters). It should be noted that, in the case of management of competencies for the design of managerial strategies and projects, the study plan contemplates the development of leadership and autonomous and interprofessional decision-making for individual and collective health care ^( 40)^ . 

The sample was non-probabilistic and consisted of 103 students chosen for convenience; 29 belonged to the Experimental Group, selected by means the habitual-historical allocation of a trained professor, with experience to teach the Care Management (administrator or manager of Nursing services) academic discipline, trained in the PBL methodology (at least 2 courses) and with a minimum of 3 years of teaching experience in the discipline; as well as 74 students distributed into 3 groups that belonged to the Control Group, in charge of professors with experience in care management (administrator or manager of Nursing services), without experience or training in PBL, who wished to participate in the study, who did not use cases in the program as a teaching method, and with a minimum of 3 years of teaching experience in the discipline.

Initially, the sample consisted of 54 students belonging to the Experimental Group (Group 1=29; Group 2=25) and 49 students from the Control Group (Group 3=33; Group 4=16); However, the group made up of 25 students belonging to the Intervention Group did not have any teacher trained in the PBL methodology to be able to implement the intervention proposed, which is why these students became part of the Control Group. However, the groups (Experimental and Control) were homogeneous (baseline measurement) for their comparison in the current study (p>0.05).

### Selection criteria

The eligibility criteria corresponded to students enrolled in the Care Management program, who completed the intervention (solution of 4 problem-scenarios) and with 90% attendance to the program. The exclusion criteria corresponded to students missing classes when the problem-scenarios were solved, as well as to those who dropped out from the program.

### Study variables

The independent variable was the implementation and solution of 4 problem-scenarios based on the PBL methodology, conceptualized as “Learning that results from the process of working towards understanding or solving a problem” ^( 20)^ . 

The dependent or result variable corresponded to the Care Management Skills: The set of interpersonal, use of the information, analytical and action-related skills ^( 9)^ , developed by the students attending the seventh semester in the Care Management academic discipline from the Bachelor’s Program in Nursing (pre- and post-test). 

The covariates were the demographic characteristics (age, gender, work, if work is related to Nursing) and the academic ones (shift, general mean, grade in the preceding discipline: “Management of Health and Nursing Services”).

### Instrument used

The Self-Assessment Instrument for Care Management Skills was designed according to the skills described ^( 6)^ . The instrument had two parts, the first with variables identifying the students (ID number, name and group), as well as sociodemographic and academic data; all in scalar, ordinal and nominal measuring scales. In addition to asking, “Have you had any experience in solving problems with the PBL method?”, with a dichotomous answer option. It is worth noting that the sociodemographic and academic variables were not asked in the post-test. 

The second section consists of 29 items divided into 4 dimensions: interpersonal skills: those that the student must develop in relation to leadership, communication and interpersonal relationships, with 10 items; skills in use of the information: those that the student must develop in relation to being creative in the search, collection and analysis of information from their environment to solve problems, with 6 item; analytical skills: those that the student must develop in relation to having a strategic vision, using statistical tools and making use of technology, with 6 items; and action-related skills: those that the student must develop in relation to being able to establish, organize and evaluate goals/objectives and making timely and risk-free decisions, with 7 items.

All the items were measured with a Likert-type scale containing the following answer options: 1=Never, 2=A few times, 3=Occasionally, 4=Almost always, and 5=Always. Each of the items was added up by dimensions and the mean values of the global skills score and by dimensions were obtained, both in the pre-test and in the post-test.

Prior to its application, content validity was performed by 3 experts in topics related to quality, management models and education. They evaluated the clarity, comprehension and relevance of each one of the questions and the validity coefficient proposed by Hernández-Nieto was calculated ^( 41)^ , obtaining a total validity of 0.87 (good to excellent coefficient). 

After having applied the instrument, Cronbach’s Alpha was calculated, obtaining a reliability of 0.91 (Excellent). Such application was useful to observe viability of the application and the students’ and researchers’ understanding of the items.

### Problem-Based Intervention, remote modality

Phase 1: 4 problem-scenarios were designed according to the best evidence available, books and current experience of professionals working as directors and Nursing managers, supervisors and heads. Each problem-scenario was designed according to the thematic content and in accordance with the learning objectives of each of the 4 thematic units from the Care Management program of the seventh semester (7/8) of the ENEO Bachelor’s Degree in Nursing ^( 40)^ . 

Likewise, the 4 scenarios were subjected to a quality evaluation by 6 experts internal and external to ENEO/UNAM in the field of management, Nursing education and PBL, with the help of the “PBL Problem Assessment Instrument” validated and with proven reliability in health sciences in Mexico, with a Cronbach’s alpha of 0.97 ^( 42)^ . The experts sent the quality assessment instruments for each of the problem-scenarios and the content validity coefficient was calculated ^( 41)^ , obtaining the following results: scenario 1, 0.78 (acceptable); scenario 2, 0.86 (good); scenario 3, 0.92 (excellent); and scenario 4, 0.92 (excellent). It is worth noting that each problem-scenario was improved according to the experts’ evaluations and comments. 

Phase 2: pre-test; during the first session in the remote modality (framing of the academic discipline) through the zoom app (remote modality), the informed consent was explained to the participants (Experimental Group and Control Group) and they were invited to participate in the research project. After having signed and/or recorded the due consent, they were provided the self-evaluation instrument for the Care Management skills via the Google Forms app. Each of the students answered the instrument with the help of their cell phones or laptop, whereas the lead researcher monitored the filling in process.

**Figure 1:**
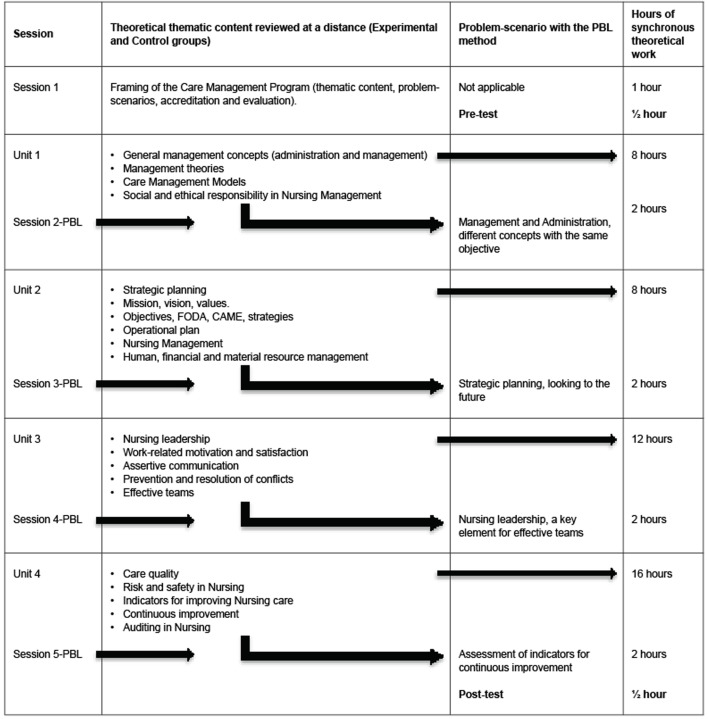
Scheme corresponding to the implementation of problem-scenarios with the PBL method. Tlalpan, CDMX, Mexico, 2021

Phase 3: implementation; in the Experimental Group and at the end of the review of the thematic content from each theoretical unit (4 units) of the Care Management Discipline ( Figure 1), the respective PBL scenario was implemented and solved (120 minutes) via zoom, where the teacher guided the students in its solution based on the PBL steps determined by the McMaster University ^( 20)^ : a) clarify unknown terms and concepts in the problem description (a brainstorming session was conducted) with the help of the Google Padlet platform; b) define the problem(s) (individually and in teams); c) analyze the problem and try to produce as many different explanations for the it as possible using prior knowledge and common sense; d) criticize the explanations proposed and try to produce a coherent description of the processes underlying the problem; e) formulate learning problems for self-directed learning; f) fill in the gaps in one’s own knowledge through self-study; and g) share the findings with the group and try to integrate the knowledge acquired into a comprehensive explanation of the problem. 

Each step and session were guided by a Facilitator Manual, in order to standardize the way to teach the problem-scenarios with the PBL method according to the steps indicated.

It is important to point out that the thematic content in both groups (Control and Experimental) was developed with different didactic strategies such as guiding questions, discussion forums, infographics, comparative charts and analogies, among others.

Phase 4: after having implemented and solved all four scenarios, the Experimental Group students self-assessed their Care Management skills a second time. For the Control Group students, at the end of the 4 thematic units reviewed, taught regularly and without the PBL methodology, each professor applied the second self-assessment of their skills developed during the Care Management academic discipline, both applied through the Google Forms app. Each of the students answered the instrument with the help of their cell phones or laptop, whereas the lead researcher monitored the filling in process.

### Data collection

The instruments were collected in 2021 by the professors in charge of the groups assigned, during the 2022- 1 period determined by the National Autonomous University of Mexico ( *Universidad Nacional Autónoma de México*, UNAM). The data were monitored by the lead researcher and they were transferred to a database in charge of a second researcher (to minimize errors in data typing). Quality of the data was ensured by using auditable algorithms to systematize and automate identification of possible errors in the characteristics recorded. In order to safeguard the participants’ confidentiality at all moments, all the records were identified by means of a single code to identify each participant, containing no personal identifiers. Access to the data collected from the participants was limited to the main researcher. The dataset analyzed in this study is available to reviewers upon formal request. 

### Data analysis

For data analysis, according to the participants’ demographic and academic characteristics, central tendency and dispersion measures (mean values, 95% confidence intervals) were used, as well as percentages. To determine homogeneity of the groups (Control and Experimental), inferential tests such as Student’s t, chi-square or Fisher’s exact test were performed, as well as Student’s t for homogeneity of the baseline measurement of the pretest for both groups. Likewise, the Kolmogorov-Smirnoff test was performed to determine data normality (p>0.05), so parametric tests were used: Student’s t test to analyze the pre-test and post-test scores of each of the groups; and the paired t test to compare the pre-test and post-test scores. Finally, univariate and multivariate linear regression was employed to analyze the possible covariates that are associated with the result (post-test). Such tests were performed in the Statistics Package for the Social Sciences program, version 25 (two-tailed, p<0.05).

### Ethical considerations

According to the ethical principles set forth in the Declaration of Helsinki, the informed consent was obtained from each of the students surveyed. The research protocol was approved by the Ethics and Research Committee of the UNAM National Nursing and Obstetrics School during 2021. It was considered a low-risk research study.

## Results

### Participants

No statistically significant differences were identified between the students from the control and experimental groups in terms of their demographic characteristics (p>0.05). In relation to their academic variables, differences were found in shift, the mean in the Bachelor’s course, and grade obtained in the preceding discipline (p<0.05) ( Table 1).

**Table 1:** Demographic and academic characteristics of the students attending the seventh semester. Tlalpan, CDMX, Mexico, 2021

	Total (n=103)	Intervention (n=29)		Control (n=74)		p-value
	Mean %	n	Mean %	n	Mean %	
**Sociodemographic**						
Age (years old), mean	22.89	29	23.07	74	22.82	0.726
Gender						
Male	71.7	24	82.8	50	67.6	0.123
Female	28.3	5	17.2	24	32.4	
Currently working						
Yes	39.8	9	31.0	32	43.2	0.181
No	60.2	20	69.0	42	71.8	
Work related to Nursing (n=41)						
Yes	36.5	2	6.9	13	17.6	0.167
No	63.5	27	93.1	61	82.4	
**Academic**						
Shift						
Morning	44.7	29	100.0	17	23.0	0.000*
Afternoon	55.3	0	0.0	57	77.0	
Mean in the Bachelor’s course	8.81	29	8.97	74	8.75	0.014†
Grade in Management (sixth semester), mean	9.36	29	9.64	74	9.26	0.007‡
Type of student						
Regular	9.7	2	6.9	8	9.7	0.546
Irregular	90.3	27	93.1	66	90.3	
Academic performance						
Very bad	1.0	0	0.0	1	1.4	0.471
Bad	1.0	0	0.0	1	1.4	
Regular	21.4	4	13.8	18	24.3	
Good	66.0	20	69.0	48	64.9	
Very good	10.7	5	17.2	6	8.1	
Academic experience in PBL						
Yes	34.0	12	41.4	23	31.1	0.187
No	24.3	9	31.0	16	21.6	
Unaware	41.7	8	27.6	35	47.3	

* Chi-square

†,‡ Student’s t

### Care Management Skills

Regarding the baseline measurement of the mean corresponding to the Care Management skills obtained by the participants (pre-test), no statistically significant differences were found in the intervention and control groups (p=0.556). No differences were either found in the dimensions: interpersonal, use of the information, analytical and action related skills (p>0.05), thus being homogeneous ( Table 2).

**Table 2:** Care Management skills in undergraduate students (pre-test) in the intervention and control groups. Tlalpan, CDMX, Mexico, 2021

	Total (n=103)	Intervention (n=29)	Control (n=74)	p-value*
	Mean value [95% Confidence Interval]			
**Global score in Care Management Skills**	4.12 [4.04-4.21]	4.09 [3.94-4.23]	4.14 [4.14-4.24]	0.556
Dimensions				
Interpersonal	4.27 [4.18-4.35]	4.30 [4.15-4.45]	4.26 [4.15-4.36]	0.651
Use of the information	4.21 [4.11-4.30]	4.14 [3.96-4.33]	4.23 [4.12-4.34]	0.410
Analytical	3.91 [3.80-4.03]	3.86 [3.64-4.08]	3.93 [3.80-4.07]	0.553
Action-related	4.03 [3.91-4.15]	3.93 [4.15-4.45]	4.07 [3.93-4.22]	0.273

* Student’s t

Before the intervention, it can be seen that the least developed skills in the students correspond to Analytics with 3.86 [3.64-4.08] *vs.* 3.93 [3.80-4.07] and to Action-related with 3.93 [4.15-4.45] *vs.* 4.07 [3.93-4.22], followed by Use of information with 4.30 [4.15-4.45] *vs.* 4.26 [4.15-4.36] and, finally, Interpersonal with 4.30 [4.15-4.45] *vs.* 4.26 [4.15-4.36]. 

Table 3 shows the results of the comparison of mean values in Care Management skills and their dimensions in the intervention and control groups in the post-test. The Intervention Group presents higher mean values in the global Care Management Skills score, when compared to the Control Group (4.23 [4,16-4.42] *vs.* 4.12 [4.10-4.32]; p=0.005). The same happened in the dimensions corresponding to Analytical (4.05 [3.89-4.29] *vs.* 3.91 [3.89-4.16]; p=0.016) and Action-related (4.12 [4.04-4.35] *vs.* 4.03 [4.07-4.33]; p=0.005) skills. The results show an improvement in the development of Care Management skills by resorting to Problem-Based learning in undergraduate Nursing students. 

**Table 3:** Care Management skills in undergraduate students (post-test) in the intervention and control groups. Tlalpan, CDMX, Mexico, 2021

	Total (n=103)	Intervention (n=29)	Control (n=74)	p-value*
	Mean value [95% Confidence Interval]			
**Global score in Care Management Skills**	4.23 [4.14-4.32]	4.23 [4.16-4.42]	4.12 [4.10-4.32]	0.005
Dimensions				
Interpersonal	4.34 [4.25-4.43]	4.34 [4.32-4.58]	4.27 [4.18-4.42]	0.085
Use of the information	4.28 [4.17-4.39]	4.28 [4.19-4.49]	4.21 [4.12-4.39]	0.146
Analytical	4.05 [3.94-4.16]	4.05 [3.89-4.29]	3.91 [3.89-4.16]	0.016
Action-related	4.20 [4.09-4.30]	4.12 [4.04-4.35]	4.03 [4.07-4.33]	0.005

* Paired t

In the multivariate linear regression analysis ( Table 4) with the total score of Care Management skills as the outcome variable after the use of Problem-Based Learning intervention, this proved to be associated with Type of student (regular-irregular), Academic performance (from excellent to poor) and Experience in PBL (r ^2^=0.270, p<0.05). 

**Table 4:** Lineal and multivariate regression models with the total score of the Care Management Skills as result variable. Tlalpan, CDMX, Mexico, 2021

Variables	Univariate lineal regression		Multivariate lineal regression	
	b	p	b	p*
**Sociodemographic**				
Age (years old)	-0.051	0.608		
Gender	-0.220	0.023		
Bachelor’s course of origin	-0.077	0.439		
Currently working	-0.033	0.738		
Work related to Nursing	-0.050	0.619		
**Academic**				
Shift	-0.168	0.090		
Mean in the Bachelor’s course	0.070	0.484		
Grade in Management of Health and Nursing Services	0.180	0.070		
Type of student	0.340	0.000	0.315	0.002
Academic performance	0.277	0.005	0.250	0.019
Experience in PBL	-0.187	0.059	-0.220	0.026

* r2 = 0,270, p<0,05

## Discussion

Care Management skills are essential in students and professionals for their professional practice in their role as managers, becoming an integral component for the change process within health institutions and Nursing services. The analysis of the data collected indicated that using scenarios with the PBL method improves care management skills in undergraduate students attending the seventh semester of the Bachelor’s Degree in Nursing. The preliminary results of the current study confirm that PBL is an effective educational strategy in the remote modality.

Previous studies have shown that the skills that a Nursing manager or administrator should have are leadership and communication ^( 43- 44)^ . Therefore, it is indispensable that professor teach and guide with significant didactic strategies ^( 19, 26- 27)^ . However, skills such as leadership, teamwork and communication, as part of the Interpersonal skills dimension, did not present changes when using scenarios with the Problem-Based Learning method in this study. This could be due to the fact that development of these skills depends to a great extent on the interaction between students and teachers, which is why the remote learning modality might have affected the fact that there were no significant differences ^( 45)^ , thus suggesting to encourage active participation of the students in digital spaces. 

Likewise, it has been documented that nurse-managers must have the ability to be creative when looking for information, its collection and especially critical analysis to make informed decisions ^( 6)^ with the purpose of improving the patients’ health. Similar studies documented that PBL increases the ability to acquire independent information in undergraduate students when compared to traditional teaching methods ^( 34, 46)^ ; however, in our findings, use of the information did not have significant changes in the Nursing students. This can be attributed to the fact that students with two years of experience in remote education (due to the COVID-19 pandemic) learned to study autonomously and independently through documentary research. 

Likewise, the results of our study in the pilot phase proved to improve the analytical skills. Similarly, previous studies have reported that problem-solving improves the analytical and critical thinking skills ^( 25- 26, 33)^ , which is in line with this research. This ability is related to the students’ vision of being strategic in solving complex managerial problems in hospital settings, using technologies and employing tools for statistical analysis; however, there are few current studies that help to be conclusive about these results. 

Finally, the action-related skills were also improved. These skills are manifested in the students when making decisions in the face of a particular situation or problem, whether real or fictitious; in other words, it is an eligible process of an option, taking into account the risks and benefits ^( 35, 47- 49)^ . In this study, implementing PBL improved the ability to plan, organize and evaluate organizational objectives and goals. Such ability is an indispensable and fundamental requirement in undergraduate Nursing curricula for the training and professional practice of future nurses. 

It is important to mention that, over time, different experiences have been published on the development of care management skills, although focused on Graduate Nursing programs in terms of administration and management, under the Problem-Based Learning method ^( 24, 50)^ , with few of them focused on undergraduate students. That is why Bachelor’s programs must begin with the development of these fundamental skills (interpersonal, use of the information, analytical and action-related) for students to be able to assume the role of managers or administrators in Health and Nursing services, thus contributing to improving care quality, medical assistance and the health of the population. 

The results of our study suggest that being a regular student, perceiving oneself with excellent academic performance, and having experience in PBL during their academic training are predictors for the development of care management skills in the remote modality; therefore, future studies should control these variables to ensure that the result is derived from using PBL in undergraduate Nursing students.

Finally, although the results are in line with the literature and show the efficacy of PBL in the development of care management skills, this study does present limitations. For being a pilot study, its findings cannot be consistent due to the type of convenience sampling and the total sample, nor can they be generalized because it was carried out only in one Nursing educational institution from Mexico. In addition, the data obtained from a self-reported assessment instrument might impose memory biases or measuring errors.

Therefore, it is suggested to generate more research to implement Problem-Based Learning in undergraduate and graduate Nursing management or administration programs, with probabilistic samples so that the results are conclusive on the development of specific Care Management skills and that they can be useful as a guide to strengthen the teaching-learning methodologies of the programs and study plans in Mexico. Likewise, the integration of new Nursing professionals to roles of managers or administrators in Nursing services should begin with continuous education using such learning strategy, in order to generate skills to carry out effective and quality Care Management for the patients.

## Conclusion

It is concluded that there is a positive effect on Care Management skills in Nursing undergraduate students with the problem-based teaching-learning method in distance mode. The students showed improvements in analytical and action-related skills; thus, PBL is considered as a powerful strategy to prepare students to assume the role of Nursing managers in their academic and professional practice. The preliminary results, as well as the identification of their strengths and weaknesses, suggest that the professors should implement this methodology for effective education for the professional world; however, in order to implement problem-based learning in remote education, teachers must be trained to effectively guide students and provide feedback for better coordination.

## References

[1] Zarate-Grajales RA (2004). La gestión del cuidado de enfermería. Index Enferm [Internet].

[2] Mororó DDS, Cruz B, Carvalho AL, Braz CM, Paiva RM (2017). Concept analysis of nursing care management in the hospital context. ACTA Paul Enferm.

[3] Kérouac S, Pepin J, Ducharme F, Duquette A, Major F (1996). El pensamiento enfermero.

[4] Christovam BP, Porto IS, Oliveira DC (2012). Nursing care management in hospital settings: The building of a construct. Rev Esc Enferm.

[5] Benner P (1982). From novice to expert. Am J Nurs.

[6] Slipicevic O, Masic I (2012). Management knowledge and skills required in the health care system of the Federation Bosnia and Herzegovina. Materia Socio Medica.

[7] Treviso P, Capeletti S, Dartora A, Alves A (2017). Competências do enfermeiro na gestão do cuidado. Rev Admin Saúde.

[8] Luther B, Barra J, Martial MA (2019). Essential nursing care management and coordination roles and responsibilities: A content analysis. Prof Case Manag.

[9] Kantanen K, Kaunonen M, Helminen M, Souminen T (2017). Leadership and management competencies of head nurses and directors of nursing in Finnish social and health care. J Res Nurs.

[10] Prieto-Rodríguez M, Suess A, March-Cerdá JC (2005). De gestoras de recursos a gestoras de cuidados: opiniones y expectativas de las supervisoras sobre su nuevo rol profesional. Enfermería Clin.

[11] Lentz S, Brenda L (2017). Nursing Care Management: Influence on bundled payments. Orthoped Nurs.

[12] Lau R, Cross W, Moss C, Campbell A, Castro M, Oxley V (2014). Leadership and management skills of general practice nurses: experience or education?. Int J Nurs Pract.

[13] Soares MI, Henriques SH, Rodriguez ZM, Souza F (2016). Saberes gerenciais do enfermeiro no contexto hospitalar. Rev Bras Enferm.

[14] Souza M, Melo C (2009). Atuação de enfermeiras nas macrofunções gestoras em saúde. Rev Enferm UERJ [Internet].

[15] Pimienta J (2012). Estrategias de enseñanza-aprendizaje [Internet].

[16] Rounds LR, Rappaport BA (2008). The successful use of: Problem-based Learning in an Online Nurse Practitioner Course. Nurs Educ Perspect [Internet].

[17] Sayyah M, Shirbandi K, Saki-Malehi A, Rahim F (2017). Use of a problem-based learning teaching model for undergraduate medical and nursing education: a systematic review and meta-analysis. Adv Med Educ Pract.

[18] Wosinski J, Belcher A, Dürrebberger Y, Anne-Claude A, Stormacq C, Gerson L (2017). Facilitating problem-based learning among undergraduate nursing students: A qualitative systematic review. Nurse Educ Today.

[19] Cartwright P, Bruce J, McInerney P (2016). Effects of problem-based learning on nurse competence: A systematic review. J Nurs Educ Pract.

[20] Barrows H, Tamblyn R (1980). Problem-based learning : an approach to medical education [Internet].

[21] Barriga D, Rojas G (2012). Estrategias docentes para un aprendizaje significativo. Una interpretación constructivista [Internet].

[22] Shin IS, Kim JH (2013). The effect of problem-based learning in nursing education: A meta-analysis. Adv Health Sci Educ.

[23] Compton RM, Owilli AO, Norlin EE, Hubbard N (2020). Does problem-based learning in Nursing Education Empower Learning?’. Nurse Educ Pract.

[24] Rideout E (2001). Transforming nursing education through Problem-Based Learning.

[25] Vargas R, Wall ML, Peres AM (2012). The problematization method applied to the subject nursing administration. Invest Educ Enferm [Internet].

[26] Park KO, Kim JK (2019). Experience of nursing management practice in graduate nurses. J Korean Acad Nurs Adm.

[27] Lin WT, Lin SY, Chou FH, Wu LM, Lee BO (2019). The longitudinal learning outcomes of using different teaching sequences in a nursing administration project. J Nurs Manag.

[28] Dellaroza M, Nakaya C, Lourenco MC, Oliveira MT, Gomes V (2015). The teaching of nursing management in undergraduate: an integrative review. Semina Ciênc Biol Saúde.

[29] Kaiser DE, Dall’Agnol CM (2017). Teaching and learning nursing management in the hospital context: An approach in the light of Pichon-Rivière. Rev Escola Enferm.

[30] Sade PM, Peres AM (2015). Development of nursing management competencies: Guidelines for continuous education services. Rev Escola Enferm.

[31] Park M (2013). Implementation of Evidence Based Nursing Education into Nursing Management Clinical Practicum: Outcome Evaluation and Diffusion Strategies. J Korean Academy Nurs Admin.

[32] Goktepe N, Turkmen E, Badir A, Hayta O (2018). Development of managerial competencies for first-level nurse managers in Turkey. Int J Caring Sci [Internet].

[33] Nkosi Z, Pillay P, Nokes KM (2013). Implementing case-based teaching strategies in a decentralised nursing management programme in South Africa. Curationis.

[34] Ancel G (2016). Problem-Solving Training: effects on the problem-solving skills and self-efficacy of nursing students. Eurasian J Educ Res.

[35] Jang KS, Park SJ (2012). Effects of action learning approaches on learning outcomes in nursing management courses. J Korean Acad Nurs Admin.

[36] Benítez-Chavira LA, Zárate-Grajales RA, Nidenda G (2021). Estrategias de enseñanza aprendizaje en gestión del cuidado de enfermería. Una revisión narrativa. Rev Enfermería Univ.

[37] Campbell DT, Stanley JC (2012). Diseños experimentales y cuasiexperimentales en la investigacion social.

[38] Rogers J, Revesz A (2019). Experimental and quasi-experimental designs.

[39] Gersten R, Fuchs LS, Compton D (2005). Quality Indicators for Group Experimental and Quasi-Experimental Research in Special Education. Except Child.

[40] Escuela Nacional de Enfermería y Obstetricia (2011). Plan de estudios de la Licenciatura de Enfermería.

[41] Nieto Hernández H (2002). Contributions to statistical analysis: the coefficients of proportional variance, content validity an Kappa.

[42] Carolina R, García Rivera RC, Gonzalez A (2019). Calidad de los problemas de ABP. Evidencia de validez de un instrumento. Investig Educ Médica.

[43] Huber D (2010). Leadership and nursing care management.

[44] Soto-Fuentes P, Reynaldos-Santana K, Martínez-Santana D, Jerez-Yáñez O (2014). Competencias para la enfermera/o en el ámbito de gestión y administración: Desafíos actuales de la profesión. Aquichan.

[45] Aslan A (2021). Problem-based learning in live online classes: Learning achievement, problem-solving skill, communication skill, and interaction. Comput Educ.

[46] Zheng S, Zhang M, Zhao C, Wang H, Sun D, Xu J (2021). The effect of PBL combined with comparative nursing rounds on the teaching of nursing for traumatology. Am J Translational Res [Internet].

[47] Thabet M, El-Sayed E, Ahmed S, Radman S (2017). The effect of problem-based learning on nursing students’ decision making skills and styles. J Nurs Educ Pract.

[48] Dowding D, Gurbutt R, Murphy M, Lascelles M, Pearman A, Summers B (2012). Conceptualising decision making in nursing education. J Res Nurs.

[49] Keshk LI, Qalawa S, El-Zaim SA (2016). Efficiency of Problem Based Learning Course at College of Nursing in Egypt and KSA: Comparative Study. Am J Educ Res.

[50] Baker C, Pesut D, McDaniel A, Fisher ML (2007). Evaluating the Impact of Problem-Based Learning on Learning Styles of Master’s Students in Nursing Administration. J Prof Nurs.

